# Silver Nanoparticles Produced by Rooibos Kombucha Suppress Bacterial Biofilms and Improve Survival in *Galleria mellonella* Infection Model

**DOI:** 10.3390/ijms27125274

**Published:** 2026-06-10

**Authors:** Razvan Vlad Opris, Alina Mihaela Baciu, Ioana Alina Colosi, Vlad Sever Neculicioiu, Anca Onaciu, Cristian-Silviu Moldovan, Ana-Maria Vlase, Carmen Costache, Adrian Florea

**Affiliations:** 1 Department of Cell & Molecular Biology, “Iuliu Hatieganu” University of Medicine and Pharmacy, 6 Louis Pasteur Street, 400349 Cluj-Napoca, Romania; razvan.opris@elearn.umfcluj.ro (R.V.O.); aflorea@umfcluj.ro (A.F.); 2 Department of Microbiology, “Iuliu Hatieganu” University of Medicine and Pharmacy, 6 Louis Pasteur Street, 400349 Cluj-Napoca, Romania; 3 Department of NanoSciences, MedFUTURE—Institute for Biomedical Research, “Iuliu Hatieganu” University of Medicine and Pharmacy, 400349 Cluj-Napoca, Romania; 4 Department of Pharmaceutical Botany, “Iuliu Hatieganu” University of Medicine and Pharmacy, 8 Victor Babeș Street, 400347 Cluj-Napoca, Romania

**Keywords:** silver nanoparticles, green biosynthesis, kombucha, rooibos (*Aspalathus linearis*), antibiofilm activity, fermented matrices, *Galleria mellonella*

## Abstract

Antibiotic resistance and biofilm-associated infections require sustainable antimicrobial platforms that combine efficacy with biocompatibility. Fermented matrices are attractive for green nanomaterial production because they provide reducing metabolites and surface-active capping compounds. Rooibos kombucha is a polyphenol-rich fermentation system with potential to serve as a biosynthetic matrix for silver nanoparticles (AgNPs). The present work aimed to develop a rooibos kombucha-enabled platform for the green biosynthesis of phytochemical-capped silver nanoparticles, AgNPs-K, and evaluate their antibacterial, antibiofilm, and *in vivo* activity. Rooibos kombucha was fermented for 14 days and profiled by liquid chromatography–tandem mass spectrometry (LC–MS/MS). AgNPs-K were generated using kombucha extract and AgNO_3_, purified, and characterized by ultraviolet–visible spectroscopy (UV–Vis), transmission electron microscopy (TEM), Fourier-transform infrared spectroscopy (FTIR), and nanoparticle tracking analysis. Antibacterial activity against eight Gram-positive and Gram-negative reference pathogens was assessed by EUCAST-based microdilution and time-kill assays. Biofilm inhibition was measured by the crystal violet assay. *In vivo* toxicity and therapeutic efficacy were evaluated in *Galleria mellonella* larvae. AgNP formation was confirmed by a surface plasmon resonance (SPR) peak at 415 nm. TEM showed predominantly spherical nanoparticles with a main size range of 20–30 nm, a hydrodynamic diameter of 98 nm, and a zeta potential of −14.62 ± 0.04 mV. AgNPs-K showed overlapping minimum inhibitory concentration and minimum bactericidal concentration values of 1.14 µg/mL for Gram-positive species and 1.33 µg/mL for Gram-negative species. Time-kill assays showed rapid bactericidal activity after threshold concentrations were reached, with sustained suppression at 24 h. Biofilm formation was abolished at 40 µg/mL and strongly reduced at lower concentrations. AgNPs-K were non-toxic up to 400 µg/mL and improved survival in six of seven infection models. Fermented rooibos kombucha functions as an effective biosynthetic matrix for the green production of phytochemical-capped AgNPs. The resulting nanoparticles combine low-dose antibacterial and antibiofilm activity with favorable *in vivo* tolerability and efficacy, supporting fermentation-enabled nanobiotechnology strategies against biofilm-associated infection.

## 1. Introduction

The escalating global prevalence of antibiotic-resistant pathogens represents one of the most critical challenges to modern medicine, threatening the effectiveness of conventional antimicrobial agents and complicating the management of infectious diseases [1]. Consequently, substantial research attention has been directed toward alternative strategies capable of overcoming microbial resistance and limiting the persistence of difficult-to-treat infections. Silver nanoparticles (AgNPs) have attracted considerable interest in this context because of their broad-spectrum antimicrobial properties and their multiple modes of action [2,3]. Beyond antimicrobial applications, silver nanomaterials spanning a wide size range, from atomically precise nanoclusters to colloidal nanoparticles, have found use in biosensing, bioimaging, catalysis, and diagnostics, reflecting the versatile physicochemical properties of nanostructured silver [2,3]. Within the antimicrobial domain specifically, recent comprehensive reviews have highlighted the multiple mechanisms through which AgNPs exert bactericidal effects, including membrane disruption, intracellular reactive oxygen species generation, and interference with essential enzyme and replication pathways [4,5]. However, concerns regarding the potential cytotoxicity and environmental persistence of AgNPs have underscored the need for synthesis methods that improve biocompatibility and reduce reliance on hazardous chemical reductants [6,7].

Recent advances in nanotechnology have increasingly focused on green synthesis routes that use natural extracts, microbial cultures, or biologically derived compounds for nanoparticle production [6]. Compared with conventional chemical methods, these approaches may reduce environmental impact while improving biocompatibility and limiting the use of hazardous reagents. In addition, biologically derived matrices are particularly attractive because they can provide both reducing molecules for metal ion conversion and stabilizing compounds that remain associated with the nanoparticle surface [7].

Kombucha tea, a fermented beverage produced by a symbiotic culture of bacteria and yeast, represents a complex and metabolically active matrix rich in organic acids, polyphenols, enzymes, vitamins, and microbial metabolites [8,9]. Despite this biochemical richness, kombucha remains insufficiently explored as a fermentation-derived platform for nanoparticle biosynthesis. Most available studies have focused on the SCOBY biomass or kombucha-associated materials in applied nanomaterial systems, including the production of nanocellulose-based constructs for tissue regeneration, and nanoparticle-enriched materials for oral implantation [10,11,12]. However, much less is known about how the liquid fermented matrix itself, with its dynamic composition of polyphenols, acids, and microbial metabolites, may influence nanoparticle formation, capping, stability, and downstream antimicrobial performance.

Given the increasing burden of biofilm-associated infections and the complexity of microbial resistance mechanisms [13], it is essential to evaluate not only nanoparticle synthesis and physicochemical characteristics, but also their functional antimicrobial performance. This includes the ability to inhibit planktonic growth and suppress biofilm development, two properties that are particularly relevant for persistent and device-associated infections. From a microbial biotechnology perspective, a biosynthetic platform is most valuable when the material it generates can be linked directly to a measurable biological effect. In the case of kombucha-mediated AgNPs, such validation requires testing against a broad panel of clinically relevant pathogens and assessing whether the resulting particles retain activity under conditions relevant to infection biology.

To extend these findings beyond in vitro screening, robust and ethically accessible *in vivo* models are needed. Among the available systems, the *Galleria mellonella* infection model has gained recognition as a reliable intermediate platform between cell-based assays and vertebrate testing [14]. Its advantages include a well-characterized innate immune response, the possibility of incubation at 37 °C, and the ability to assess toxicity, therapeutic efficacy, and survival in a rapid and reproducible manner [15,16]. These characteristics make *G. mellonella* particularly suitable for the evaluation of biosynthesized nanomaterials with antimicrobial intent.

In this context, the present study investigated fermented rooibos kombucha as a biosynthetic matrix for the green production of phytochemical-capped silver nanoparticles. By combining phytochemical profiling of the fermented matrix with nanoparticle synthesis, physicochemical characterization, antibacterial testing, antibiofilm assessment, and *in vivo* validation in *Galleria mellonella*, the study aimed to evaluate whether rooibos kombucha can function not only as a source of reducing compounds but also as a fermentation-enabled platform for generating biologically active nanomaterials with relevance for antimicrobial biotechnology.

## 2. Results

### 2.1. Kombucha Tea and Biosynthesized AgNPs-K Characteristics

**Figure 1:**
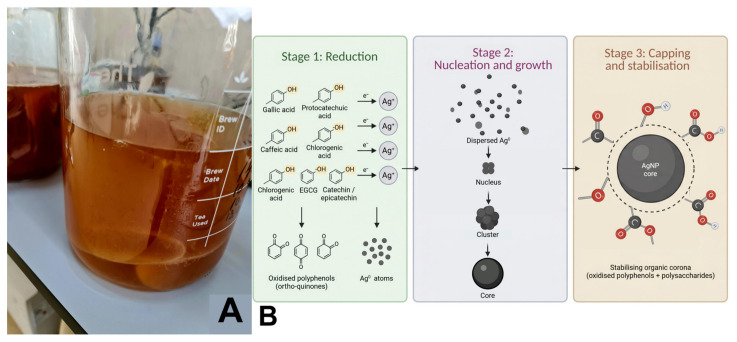
(**A**) Mature rooibos kombucha tea following two-week fermentation. (**B**) Proposed mechanism of AgNPs-K biosynthesis from fermented rooibos kombucha. (i) Catechol- and galloyl-containing phenolics from the T2 kombucha matrix donate electrons to reduce Ag^+^ to Ag^0^; (ii) Ag^0^ atoms nucleate and grow into metallic silver clusters; (iii) deprotonated carboxylate (–COO−), hydroxyl, carbonyl, and glycosidic groups from polyphenols and polysaccharides adsorb onto the nanoparticle surface, forming a stabilizing organic corona. Created in BioRender. Baciu, A. (2026) (https://BioRender.com/a6uowc5).

In order to characterize the biochemical environment responsible for the green synthesis of the silver nanoparticles, we performed HPLC analysis of the rooibos kombucha before (T0) and after 2 weeks of fermentation (T2), the latter being the tea used for AgNP synthesis (Table 1). The T2 profile showed that the kombucha contains a rich mixture of redox-active phenolic acids and flavonoids, including substantial amounts of protocatechuic acid (~12.8 µg/mL), isoquercitrin (~14.4 µg/mL), gallic acid (~7.0 µg/mL), ferulic acid (~4.6 µg/mL), EGCG (~3.9 µg/mL), rutin (~6.2 µg/mL), vitexin and hyperoside (~5–6 µg/mL), together with lower concentrations of catechin, epicatechin, p-coumaric, vanillic, syringic and luteolin. Fermentation converted part of the more complex or esterified phenolics present at T0 into simpler, more reactive phenolic acids. Compounds that were <LOQ in the starting tea, including caffeic and chlorogenic acids, became detectable at T2, and both 4-O-caffeoylquinic and gallic acid increased, indicating enzymatic hydrolysis and biotransformation by the SCOBY (Figure 1A,B). At the same time, ferulic acid, EGCG, and quercetol decreased but remained present in appreciable amounts, so that the overall pool of multi-hydroxylated and carboxylated molecules was maintained during fermentation. This phenolic landscape at T2 provided a plausible chemical basis for the nanoparticle synthesis, with abundant catechol- and galloyl-containing structures (gallic, caffeic, chlorogenic, protocatechuic acids, EGCG, catechin/epicatechin) that can act as electron donors to reduce Ag^+^ to Ag^0^. While carboxyl, carbonyl and glycosidic C–O groups could subsequently coordinate and adsorb on the silver surface, to form a stabilizing organic corona.

**Figure 2:**
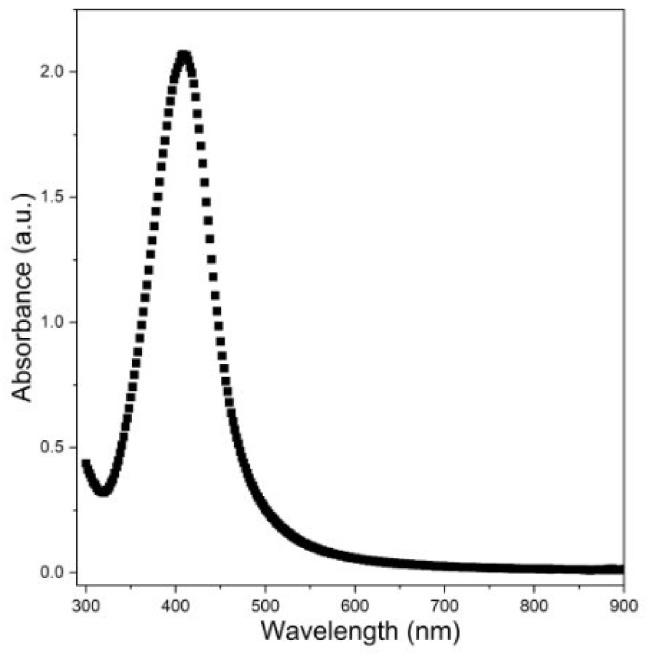
UV-Vis of kombucha biosynthesized silver nanoparticles solution.

**Figure 3:**
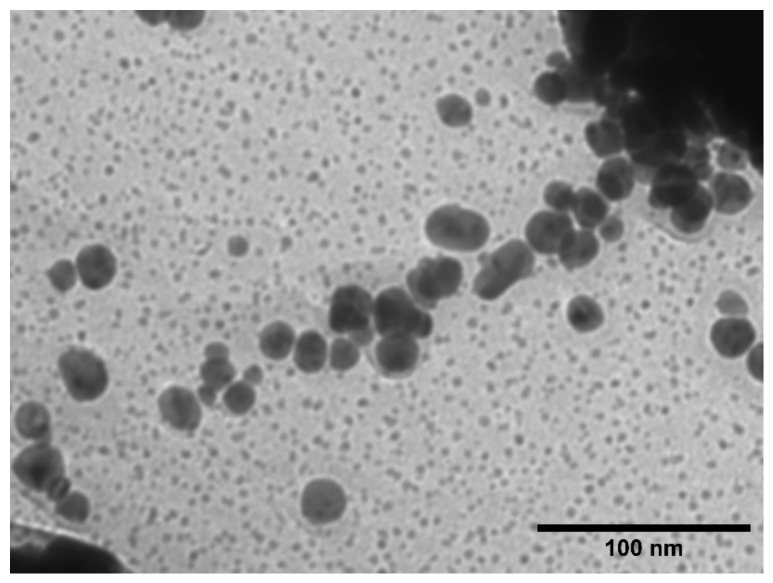
TEM image of the kombucha biosynthesized silver nanoparticles.

**Figure 4:**
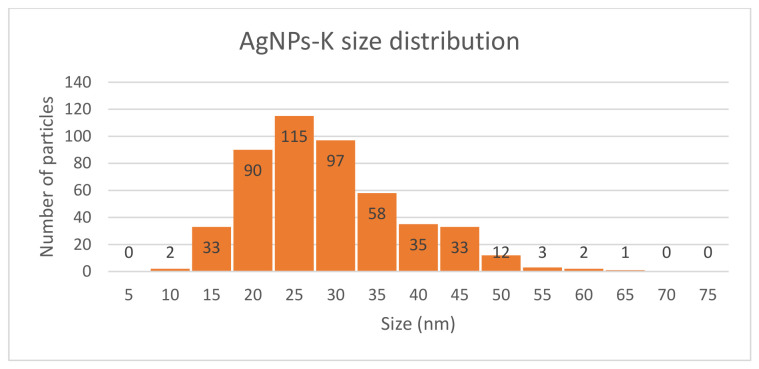
Histogram depicting the size distribution of kombucha biosynthesized silver nanoparticles.

Nanoparticle formation was detected through UV-Vis at a peak absorbance of 415 nm (Figure 2). The shape of the obtained nanoparticles was determined as spherical (Figure 3), and the size distribution showed that most obtained particles were between 20 and 30 nm (Figure 4). NTA determined the hydrodynamic size of the obtained AgNPs-K at 98 nm (94.3% of a total of 487 traced particles) and the zeta potential of −14.62 ± 0.04 mV.

**Figure 5:**
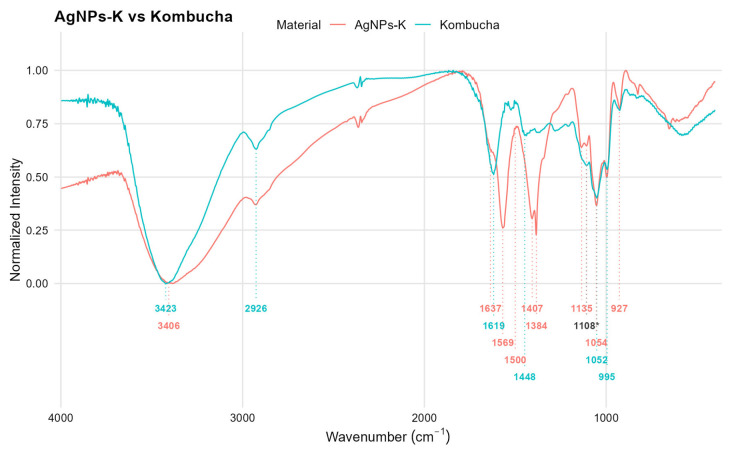
FTIR spectra of AgNPs-K (red spectra) and the fermented Kombucha tea (blue spectra) used in their synthesis. Wavenumbers are given in cm^−1^. The 1108 cm^−1^ band marked with an asterisk is preserved with similar position and intensity in both spectra.

The FTIR spectra of the rooibos kombucha and the corresponding AgNPs-K were highly similar in band pattern but showed clear shifts in key regions, confirming that the nanoparticles were coated with kombucha-derived biomolecules and that specific functional groups from the phenolic/flavonoid fraction were involved in silver reduction and capping (Figure 5). The kombucha spectrum showed a broad O–H stretching band at ~3423–3397 cm^−1^ and a C–H stretching band at ~2926 cm^−1^, consistent with abundant hydroxylated polyphenols, flavonoid glycosides and carbohydrates. In the AgNPs spectrum, this broad O–H band slightly shifted to lower wavenumbers (~3406–3386–3380 cm^−1^) and changes in intensity, which indicates stronger hydrogen bonding and/or coordination of phenolic and sugar O–H groups to the silver surface after their involvement in Ag^+^ reduction. The carbonyl region of kombucha, with bands at ~1654–1619–1624 cm^−1^, is attributed to C=O stretching of phenolic and quinic acids as well as flavonoid carbonyls and conjugated C=C, whereas in AgNPs this region is reorganized, with a new/strong band at ~1569 cm^−1^ and a shifted band around ~1637–1624 cm^−1^, suggesting that carboxyl and carbonyl groups of these compounds were partially deprotonated and coordinated to metallic silver. Likewise, the kombucha bands at ~1448–1438–1431 cm^−1^, assigned to aromatic C=C and COO^−^ symmetric stretch, move and split into bands at ~1384, ~1407 and ~1500 cm^−1^ in the AgNPs, a typical signature of carboxylate groups interacting with a metal surface. In the C–O/C–O–C region, kombucha shows intense bands around ~1108, ~1052 and ~995 cm^−1^ (plus a feature near ~925 cm^−1^) due to glycosidic C–O–C and C–O stretching of flavonoid glycosides and polysaccharides. In AgNPs, the same region is preserved but with slight shifts (bands at ~1135, ~1108, ~1054, ~996 and ~927 cm^−1^) and altered intensities, consistent with adsorption of these sugar-rich structures onto the nanoparticle surface.

### 2.2. Antimicrobial Effects

#### 2.2.1. Determination of Minimum Inhibitory Concentration and Minimum Bactericidal Concentration

**Figure 6:**
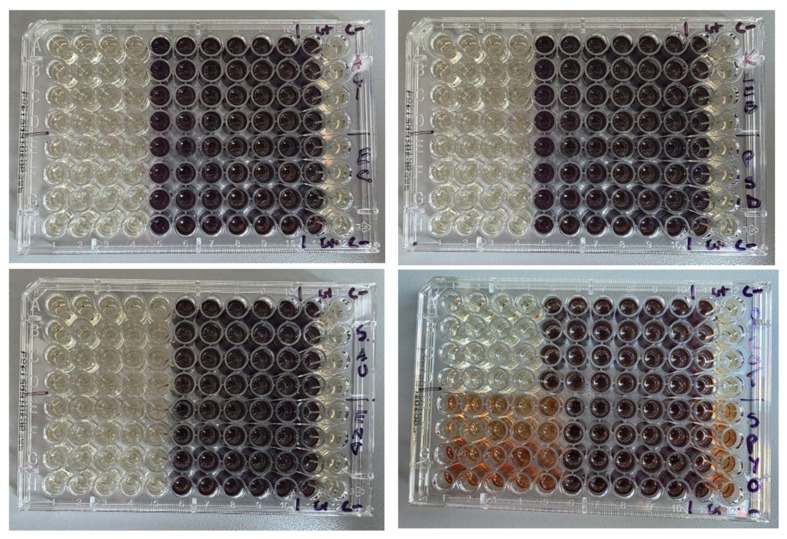
MTT assay results of AgNPs-K on tested bacterial species. Each plate was used for 2 bacterial species. Columns on plates denoting line of replicates (A, B, C, D for one bacterial isolate and E, F, G, H for the second). Rows from 1 to 10 contain increasing dilutions of AgNPs. Row 11 contained positive control (bacteria with culture media), Row 12 sterility control (culture media). Black pigmentation of wells denotes active bacterial metabolism after application of MTT.

The MIC and MBC were confirmed to overlap at a AgNP-K concentration of 1.14 µg/mL (1/350 dilution) for the tested Gram-positive bacterial strains (*Staphylococcus aureus*, *Streptococcus pyogenes*, and *Enterococcus faecium*) and 1.33 µg/mL (1/300 dilution) for Gram-negative bacteria (*Escherichia coli*, *Pseudomonas aeruginosa*, *Proteus mirabilis*, *Klebsiella pneumoniae*, and *Acinetobacter baumanii*), as evidenced by the absence of black pigment formation in the MTT assay (Figure 6) and 100% reduction in CFU count compared to control. The kombucha tea filtrate, tested in parallel across the same dilution series as AgNPs-K (1/150 to 1/1000) and against the same eight bacterial species, showed no inhibitory effect at any concentration, with OD_620_ values indistinguishable from those of the untreated positive control. This confirms that the antimicrobial activity reported below is attributable to the biosynthesized AgNPs-K and not to residual kombucha components.

#### 2.2.2. Time-to-Kill Assay

**Figure 7:**
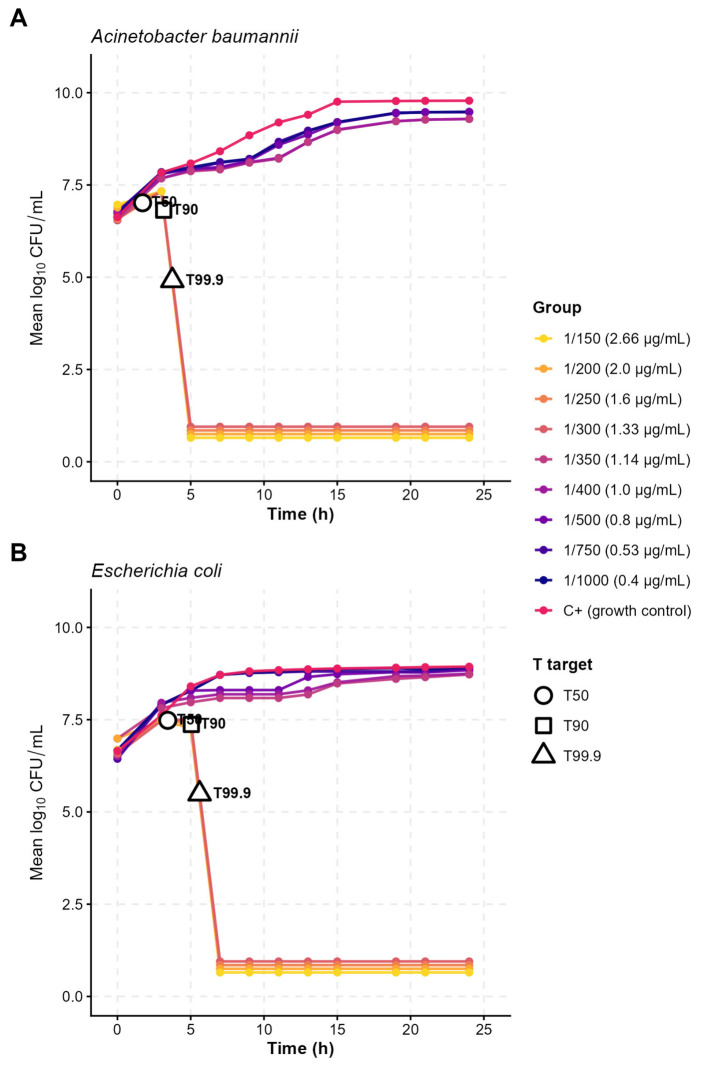
Time-to-kill kinetics of AgNPs-K against *Acinetobacter baumanii* (**A**) and *Escherichia coli* (**B**). Mean log10 CFU per mL are shown over time for cultures exposed to AgNPs K at the indicated final dilutions, with equivalent concentrations in µg per mL shown in parentheses. C+ denotes the growth control without AgNPs K. Symbols mark the estimated time to reach T50, T90, and T99.9, defined as 0.301, 1.000, and 3.000 log10 reductions versus C+ at the same time point, obtained by interpolation from the mean curves. Values are means of replicates with SD calculated but not displayed.

**Figure 8:**
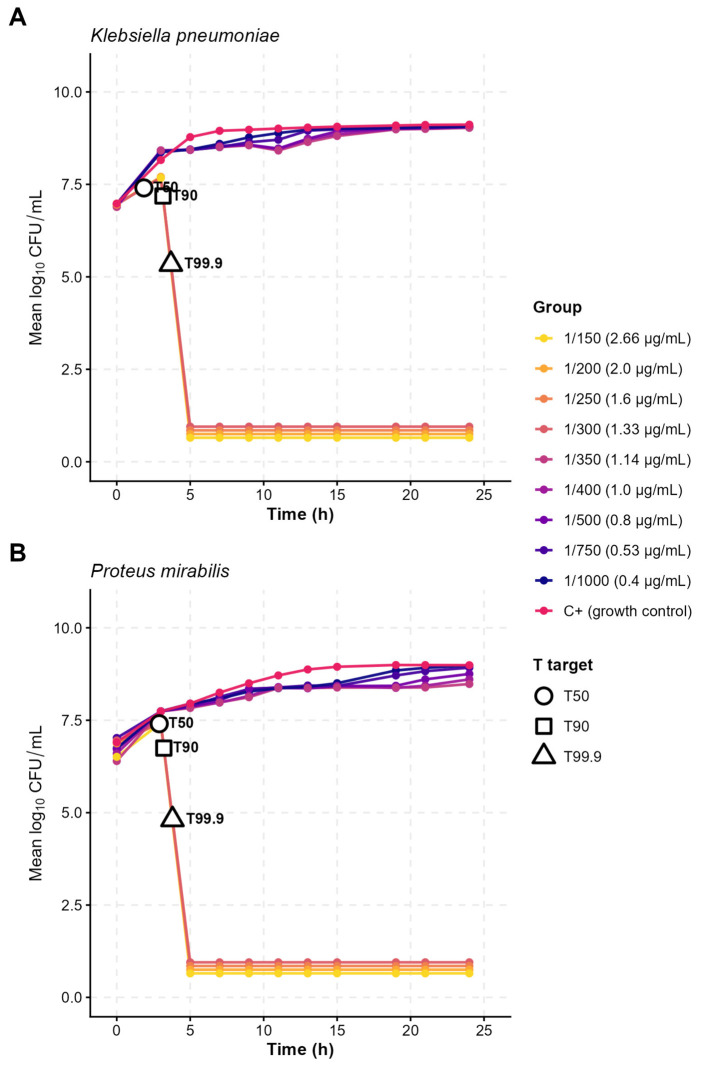
Time-to-kill kinetics of AgNPs-K against *Klebsiella pneumoniae* (**A**) and *Proteus mirabilis* (**B**). Mean log10 CFU per mL are shown over time for cultures exposed to AgNPs K at the indicated final dilutions, with equivalent concentrations in µg per mL shown in parentheses. C+ denotes the growth control without AgNPs K. Symbols mark the estimated time to reach T50, T90, and T99.9, defined as 0.301, 1.000, and 3.000 log10 reductions versus C+ at the same time point, obtained by interpolation from the mean curves. Values are means of replicates with SD calculated but not displayed.

**Figure 9:**
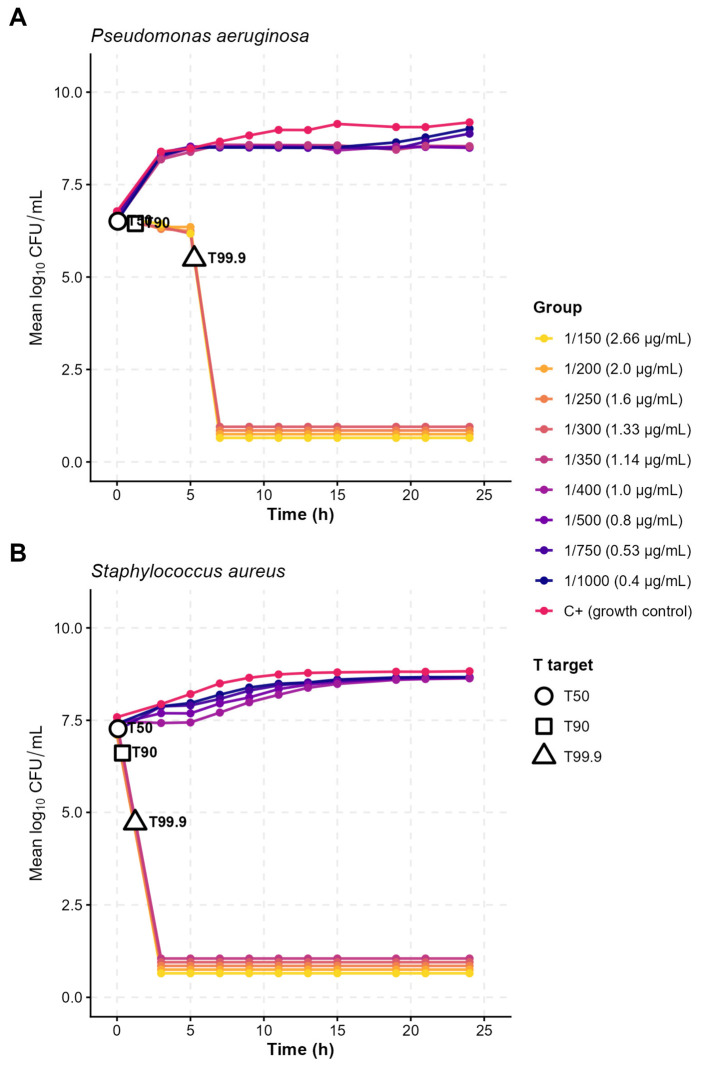
Time-to-kill kinetics of AgNPs-K against *Pseudomonas aeruginosa* (**A**) and *Staphylococcus aureus* (**B**). Mean log10 CFU per mL are shown over time for cultures exposed to AgNPs K at the indicated final dilutions, with equivalent concentrations in µg per mL shown in parentheses. C+ denotes the growth control without AgNPs K. Symbols mark the estimated time to reach T50, T90, and T99.9, defined as 0.301, 1.000, and 3.000 log10 reductions versus C+ at the same time point, obtained by interpolation from the mean curves. Values are means of replicates with SD calculated but not displayed.

**Figure 10:**
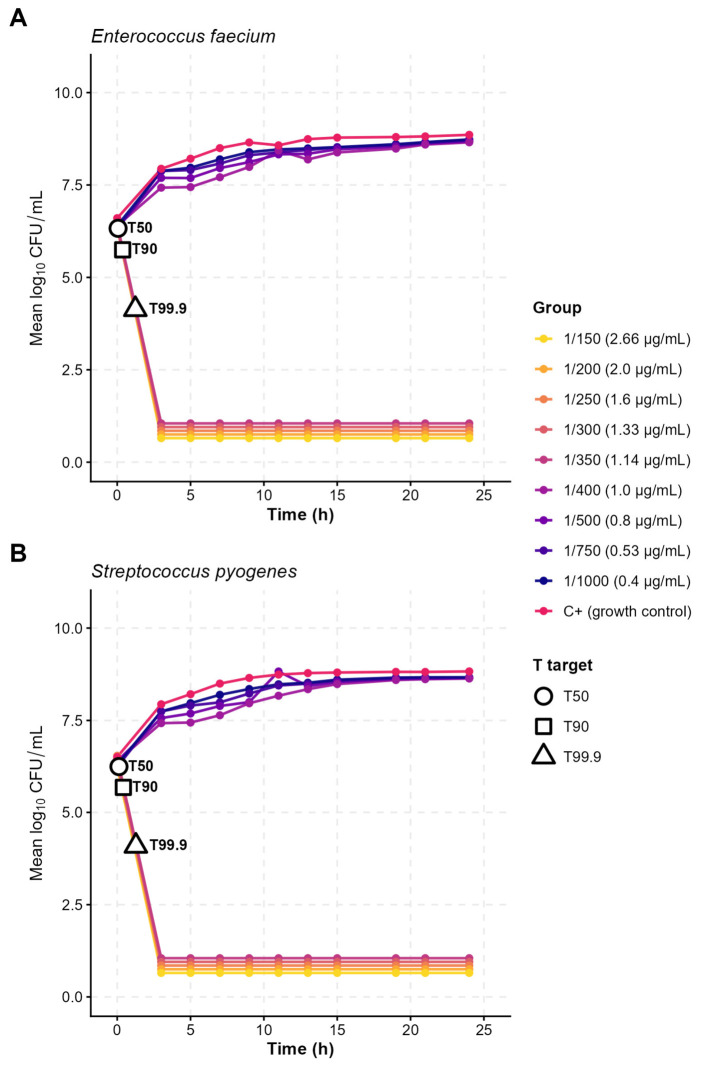
Time-to-kill kinetics of AgNPs-K against *Enterococcus faecium* (**A**) and *Streptococcus pyogenes* (**B**). Mean log10 CFU per mL are shown over time for cultures exposed to AgNPs K at the indicated final dilutions, with equivalent concentrations in µg per mL shown in parentheses. C+ denotes the growth control without AgNPs K. Symbols mark the estimated time to reach T50, T90, and T99.9, defined as 0.301, 1.000, and 3.000 log10 reductions versus C+ at the same time point, obtained by interpolation from the mean curves. Values are means of replicates with SD calculated but not displayed.

Across all eight species, K-AgNPs produced clear, time-dependent bactericidal activity once a species-specific concentration threshold was exceeded, with the corresponding kill curves for those bactericidal doses almost completely overlapping (Figure 7, Figure 8, Figure 9 and Figure 10). For Gram-negative isolates (*A. baumannii*, *E. coli*, *K. pneumoniae*, *P. mirabilis*, *P. aeruginosa*), all AgNPs-K concentrations from 1/150 to 1/300 (2.66–1.33 µg/mL) led to a rapid decrease in CFU counts. After a short initial lag, viable counts dropped from ~10^6^ CFU/mL at baseline to near or below the limit of detection by about 5 h and remained suppressed for the full 24 h period, while the growth control (C+) continued to rise to ~10^9^ CFU/mL. This corresponds to ≥3 -log_10_ (typically ≥6–7 -log_10_) reductions vs. C+ at 24 h, fulfilling the bactericidal definition for all dilutions ≥1/300. Lower concentrations (≤1/350; 1.14 µg/mL and below) did not achieve sustained bactericidal effects, with curves at 1/350–1/1000 showing similar progression to C+ or only modestly depressed, yielding Δlog_10_(24 h) values in the no net inhibition range.

For Gram-positive isolates (*S. aureus*, *S. pyogenes*, *E. faecium*), the same pattern was observed, but the bactericidal window extended down to slightly lower nominal concentrations. All dilutions ≥1/150 (2.66–1.14 µg/mL; corresponding to 1/150 through 1/350) produced a steep decline in CFU/mL to ~10^1^ or below within the first few hours, with no regrowth up to 24 h, while the controls continued to grow. Again, endpoint Δlog_10_(24 h) values were well above three logs for these doses, confirming bactericidal activity. Because the kill curves at 1/150, 1/200, 1/250, 1/300 (and, for Gram-positives, 1/350) nearly superimpose, increasing the concentration above the threshold did not substantially accelerate killing or deepen the final reduction, consistent with a threshold effect of AgNPs-K rather than a simple linear dose–response. In contrast, sub-threshold dilutions showed at best modest growth delay without achieving bactericidal criteria.

#### 2.2.3. Biofilm Quantification Essay

AgNPs-K showed a concentration-dependent anti-biofilm effect with a clear concentration threshold (Table 2). At 40 µg/mL, biofilm was not detected in any bacterial strain, and at 4 µg/mL, inhibition remained complete for all species except *K. pneumoniae*, which produced weak biofilm relative to the moderate positive control, indicating comparatively higher tolerance. Below 4 µg/mL, the inhibitory effect decreased in a species-dependent manner. *S. pyogenes* remained the most susceptible, with biofilm still absent to 1.14 µg/mL and only weak-to-moderate production at lower concentrations, not exceeding the positive-control classification. *S. aureus* also remained attenuated across the range, shifting from weak biofilm at 2–1.6 µg/mL to moderate biofilm at ≤1.14 µg/mL, without reaching strong production. *A. baumannii* and *E. coli* showed intermediate susceptibility, with complete inhibition at 2 µg/mL and predominantly weak biofilm at 1.6–0.8 µg/mL; at lower concentrations, biofilm increased toward the respective positive-control levels, with *A. baumannii* returning to strong at 0.32 µg/mL. *P. aeruginosa* exhibited incomplete suppression below 4 µg/mL, progressing from weak and moderate biofilm at 2–0.8 µg/mL to strong biofilm at ≤0.53 µg/mL, consistent with the strong positive control.

### 2.3. In Vivo Experiments Using Galleria mellonella Larvae

#### 2.3.1. Acute Toxicity of AgNPs-K in *G. melonella* Larvae

**Figure 11:**
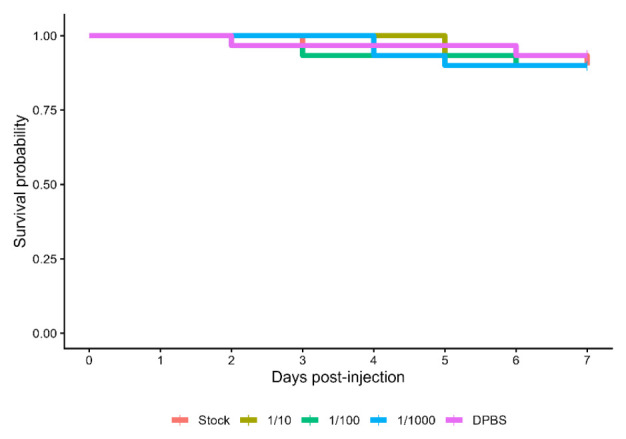
Kaplan–Meier survival of *G. mellonella* larvae exposed to different concentrations of AgNPs-K. Concentrations of AgNPs-K: stock = 400 µg/mL; 1/40 = 40 µg/mL; 1/100 = 4 µg/mL; 1/1000 = 0.4 µg/mL. DPBS used as control. N = 30 larvae per group.

The biosynthesized AgNPs were well tolerated by *Galleria mellonella* across all tested concentrations, including the undiluted stock (400 µg/mL AgNP-K), with 7-day survival being indistinguishable from the DPBS control (Figure 11).

#### 2.3.2. Therapeutic Efficacy of AgNPs-K Against Bacterial Infection in *G. melonella* Larvae

**Figure 12:**
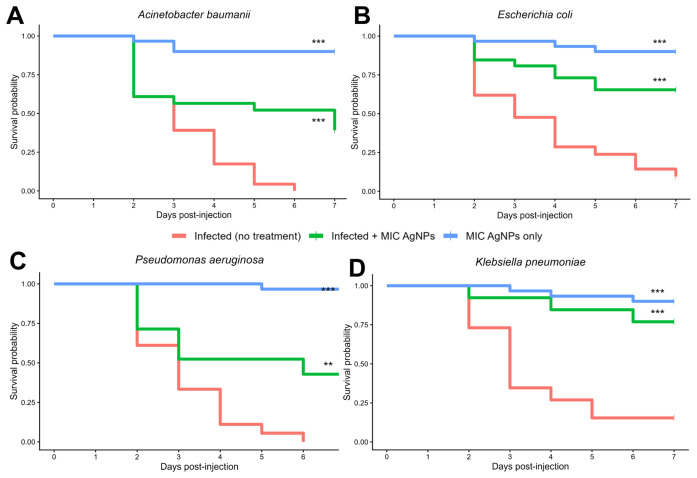
Kaplan–Meier survival of *Galleria mellonella* larvae infected with Gram-negative bacteria and treated with AgNPs-K at the MIC for each species. Panels show infection with *Acinetobacter baumannii* (**A**), *Escherichia coli* (**B**), *Pseudomonas aeruginosa* (**C**), and *Klebsiella pneumoniae* (**D**). Groups are infected with no treatment, infected plus MIC AgNPs-K, and MIC AgNPs-K only. Survival was monitored for 7 days post-infection. Asterisks indicate differences versus infected with no treatment by log rank test. ** *p* < 0.01, *** *p* < 0.001. N = 30 larvae per group.

**Figure 13:**
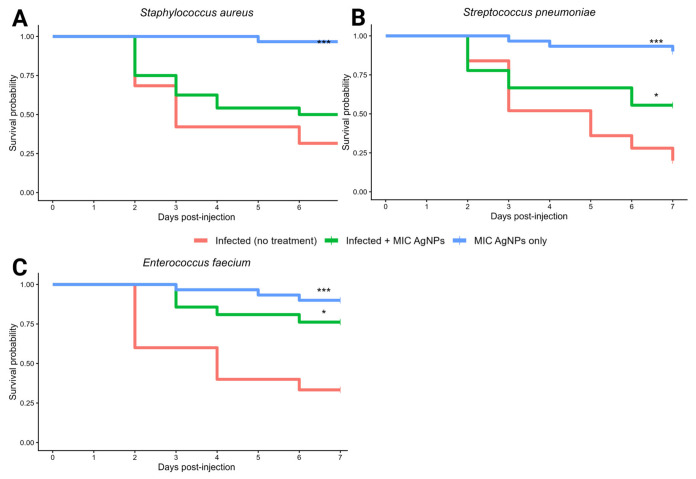
Kaplan–Meier survival of *Galleria mellonella* larvae infected with Gram-positive bacteria and treated with AgNPs-K at the MIC for each species. Panels show infection with *Staphylococcus aureus* (**A**), *Streptococcus pneumoniae* (**B**), *Enterococcus faecium* (**C**). Groups are infected with no treatment, infected plus MIC AgNPs-K, and MIC AgNPs-K only. Survival was monitored for 7 days post-infection. Asterisks indicate differences versus infected with no treatment by log-rank test. * *p* < 0.05,*** *p* < 0.001. N = 30 larvae per group.

Kaplan–Meier analysis demonstrated that infection alone markedly reduced survival, whereas treatment with AgNPs at the MIC significantly improved outcomes in most models. Holm-adjusted pairwise log-rank tests versus the infected control showed robust protection for *Acinetobacter baumannii*, *Escherichia coli*, and *Klebsiella pneumoniae* (*p* < 0.001), a significant benefit for *Pseudomonas aeruginosa* (*p* < 0.01), and modest but significant effects for the Gram-positive species *Enterococcus faecium* and *Streptococcus pneumoniae* (both *p* < 0.05). In the *Staphylococcus aureus* model, the treated curve trended higher but did not reach statistical significance after adjustment. Collectively, these results indicate that AgNPs at MIC are non-toxic in larvae and confer meaningful survival advantages in six of seven infection models, with the largest effects observed against Gram-negative bacteria (Figure 12 and Figure 13).

## 3. Discussion

In this study, fermented rooibos kombucha functioned as a biologically active matrix for the green synthesis of silver nanoparticles with strong antibacterial, antibiofilm, and *in vivo* therapeutic activity. The fermented matrix served not as a passive extract source but as both an active reducing environment and a source of phytochemical components that remained associated with the nanoparticle surface, supporting fermentation-derived systems as integrated platforms for nanomaterial generation. By combining phytochemical profiling, physicochemical characterization, antimicrobial and biofilm testing, and *in vivo* validation, this study places rooibos kombucha-mediated synthesis within a broader fermentation-enabled strategy for developing biologically active antimicrobial nanomaterials.

At the physicochemical level, the kombucha used as a reducing and capping agent contained a complex mixture of redox-active phenolic acids and flavonoids, including protocatechuic and gallic acids, isoquercitrin, rutin, vitexin, hyperoside, catechins and related derivatives. Fermentation shifted the profile toward simpler, more reactive phenolic acids, while maintaining a rich pool of multi-hydroxylated and carboxylated structures. These compounds are well-suited to act as electron donors for Ag^+^ reduction and as multidentate ligands for capping, providing a plausible mechanistic basis for nanoparticle formation and stabilization in this system. In agreement with this, NTA revealed a predominant hydrodynamic population around 98 nm with a moderately negative zeta potential of −14.62 ± 0.04 mV, consistent with individually dispersed particles coated by an organic shell rather than large aggregates. The zeta potential of −14.62 ± 0.04 mV measured for AgNPs-K falls within the range commonly reported for biogenic silver nanoparticles capped with complex plant or fermented extracts. For context, *Tridax procumbens*-derived AgNPs displayed a zeta potential of −15.3 mV with a hydrodynamic diameter of approximately 150 nm [17] while *Cucumis prophetarum*-mediated AgNPs showed −24.7 mV [18]. Values exceeding −30 mV, such as those reported for *Buchanania lanzan* leaf AgNPs (−27.6 mV) [19] or *Clinacanthus nutans* AgNPs (−42.8 to −43.9 mV) [20], are generally associated with higher electrostatic stabilization. The moderate negative charge of AgNPs-K suggests that colloidal stability in this system is maintained not solely through electrostatic repulsion but likely also through steric stabilization provided by the adsorbed organic corona of kombucha-derived polyphenols and polysaccharides, as supported by our FTIR data. This dual stabilization mechanism, combining electrostatic and steric contributions, is commonly described for biogenic nanoparticles coated with complex phytochemical mixtures [7]. The UV–Vis surface plasmon resonance peak at 415 nm and TEM images showing predominantly spherical particles in the 20–30 nm range are consistent with the spectral and morphological features reported for other biogenic silver nanoparticles synthesized using plant and fungal extracts [21]. FTIR analysis further confirmed that kombucha-derived hydroxyl, carbonyl and carboxylate groups were involved in Ag^+^ reduction and remained adsorbed on the nanoparticle surface, generating an organic corona that likely contributed to colloidal stability and biological interactions.

Comparable observations have been reported for other nanomaterials synthesized using kombucha or kombucha-derived matrices, reinforcing the idea that this fermented environment provides both reducing power and a rich source of capping ligands. For instance, Al-Kalifawi [22] synthesized silver nanoparticles using Hamza’s Khubdat kombucha tea as the sole reducing and capping agent, obtaining spherical AgNPs with an SPR band around 430 nm and an average crystallite size of ~29 nm. A related study by El-Fallal et al. [23] used kombucha extract to biosynthesize spherical ZnO nanoparticles, with a diameter of ~23 nm, and positively charged. Although direct comparison across different metals is not straightforward, their data and ours together indicate that kombucha-derived systems can reliably yield small and stable metal nanoparticles. Beyond the liquid fraction, Aboul-Nasr et.al. [24] recently integrated mycosynthesized AgNPs (14.5 nm) into kombucha SCOBY cellulose membranes to form AgNPs@KC nanocomposites (17.9 nm). Moreover, bioflocculants secreted by *Pichia kudriavzevii* isolated from kombucha SCOBY have been successfully utilized as templates for the green synthesis of silver and iron nanoparticles [10], primarily for environmental and water-treatment applications, underscoring the broader potential of kombucha-associated microbiota as nanofactories. The involvement of phenolic and polysaccharide-derived functional groups in nanoparticle capping observed here is broadly consistent with reports from other kombucha-mediated and kombucha-associated systems. Al-Kalifawi [22] reported FTIR bands attributable to hydroxyl, carbonyl, and amide groups on the surface of AgNPs synthesized using kombucha tea, suggesting that proteins and polyphenols from the fermented matrix participated in both reduction and stabilization. El-Fallal et al. [23] identified O–H, C=O, and C–O–C stretching bands in their kombucha-biosynthesized ZnO nanoparticles, which they attributed to adsorption of tea-derived phenolics and carbohydrates onto the nanoparticle surface, paralleling the spectral features observed in our AgNPs-K. Similarly, Tsilo et al. [10] reported that bioflocculants from *Pichia kudriavzevii* isolated from kombucha SCOBY, rich in hydroxyl and carboxyl functionalities, served as effective templates for AgNP capping. More recently, Aboul-Nasr et al. [24] documented hydroxyl, carbonyl, and glycosidic vibrations in AgNPs@KC nanocomposites prepared by integrating mycosynthesized AgNPs into kombucha SCOBY cellulose, confirming that kombucha-derived carbohydrate and phenolic structures contribute to the organic corona across different synthesis configurations. Taken together, these data indicate that kombucha and kombucha-associated matrices consistently provide polyphenol- and polysaccharide-based capping, with the specific profile depending on the tea substrate, fermentation conditions, and metal species.

The antimicrobial data indicate that AgNPs-K are highly potent against a panel of clinically relevant Gram-negative (*Escherichia coli*, *Pseudomonas aeruginosa*, *Proteus mirabilis*, *Klebsiella pneumoniae*, *Acinetobacter baumannii*) and Gram-positive (*Staphylococcus aureus*, *Streptococcus pyogenes*, *Enterococcus faecium*) reference strains. MIC and MBC values overlapped at 1.14 µg/mL for Gram-positive bacteria and 1.33 µg/mL for Gram-negative bacteria, with complete loss of viability at these concentrations in CFU assays. When expressed in mg/mL, these values (0.00114–0.00133 mg/mL) compare favorably with those reported for other silver-based formulations. The kombucha-biosynthesized ZnO nanoparticles developed by El-Fallal et al. [23] achieved MICs of 25–40 µg/mL against a panel of ATCC strains, including *E. coli*, *S. aureus*, *K. pneumoniae* and *P. aeruginosa*. Prasastha et al. [25] constructed nanosilver entrapped with cinnamaldehyde (AgC), using as a base AgNPs synthesized using *Lactobacillus acidophilus* that achieved MICs against MDR enteroaggregative *E. coli* (EAEC) ranged from 0.008–0.016 mg/mL, approximately one order of magnitude higher than the MICs observed for our AgNPs-K, albeit against particularly resistant clinical isolates. Biologically synthesized AgNPs produced with *Aspergillus flavus* or *Citrus latifolia* extracts [21] exhibited MICs between 4 and >128 µg/mL (0.004–0.128 mg/mL) against MDR *P. aeruginosa*, while electrochemically synthesized AgNPs [26] tested on cystic fibrosis-associated *P. aeruginosa* and *Burkholderia cepacia* typically showed MICs in the low µg/mL range (median around 1.06 and 2.125 µg/mL, respectively). Although direct numerical comparison is complicated by differences in bacterial species, strain background, inoculum, and methodology, these data collectively suggest that AgNPs-K sit at the very active end of the spectrum for silver-based nanomaterials, at least against standard ATCC strains. The observation that Gram-positive bacteria in this study were inhibited at a marginally lower AgNPs-K concentration (1.14 µg/mL) compared to Gram-negative bacteria (1.33 µg/mL) is noteworthy, as the opposite pattern is frequently reported in the AgNP literature. Several factors may contribute to this finding. First, the difference corresponds to a single dilution step in the microdilution series and is therefore modest, falling within the range of inter-experiment variability accepted by EUCAST methodology. Second, the phytochemical corona derived from the kombucha matrix may interact differently with the two envelope architectures. The outer membrane of Gram-negative bacteria presents a lipopolysaccharide barrier that can restrict initial nanoparticle contact and Ag^+^ penetration, potentially offsetting the advantage of a thinner peptidoglycan layer. In contrast, although Gram-positive bacteria possess a thicker peptidoglycan wall, the absence of an outer membrane permits more direct interaction between the phytochemical-capped nanoparticle surface and the underlying cytoplasmic membrane. Third, the teichoic and lipoteichoic acids embedded in the Gram-positive peptidoglycan carry a net negative charge and may electrostatically attract the silver cations released from the AgNPs-K surface, concentrating silver ions at the cell envelope and enhancing local antimicrobial activity [27,28]. Similar patterns of slightly greater Gram-positive susceptibility at the MIC level have been reported for phytochemical-capped AgNPs in other studies, particularly when complex organic coronas modify particle–cell interactions [29,30].

The time–kill experiments add important kinetic information to the static MIC/MBC endpoints. Once a species-specific concentration threshold was exceeded, AgNPs-K produced rapid and sustained bactericidal activity, with viable counts falling from ~10^6^ CFU/mL to near or below the detection limit within about 5 h and remaining suppressed at 24 h. Increasing the dose above this threshold did not substantially accelerate killing, consistent with a saturable or threshold-driven mechanism rather than a strictly linear dose–response, which is in line with other AgNP studies where bactericidal effects emerge once concentrations reach ≥1–2 × MIC in time–kill assays [29,31,32]. This pattern is reminiscent of the time–kill behavior of AgC, where MBC-level exposure eradicated MDR-EAEC within 120 min [25], and of other studies in which AgNPs cause a swift collapse of bacterial viability once sufficient particle–cell contact and silver ion exposure are achieved [21,26,33]. Mechanistically, the threshold effect observed here aligns well with established models in which AgNPs adhere to and penetrate the cell envelope, induce membrane damage, generate reactive oxygen species (ROS), and release Ag^+^ that interfere with thiol-containing enzymes and DNA replication [27,28,33]. Once a critical density of nanoparticles or ions accumulates at the cell surface, additional dosing may offer limited incremental benefit, a concept supported by kinetic and mechanistic analyses of AgNP action in which membrane disruption, oxidative stress and protein damage jointly drive bacterial death [27,33].

Beyond planktonic inhibition, AgNPs-K showed a pronounced anti-biofilm effect *in vitro*. At 40 µg/mL, biofilm formation was abolished in all six strains. At 4 µg/mL, it remained undetectable for *S. pyogenes*, *S. aureus*, *A. baumannii*, *E. coli*, and *P. aeruginosa*, while *K. pneumoniae* was reduced to a weak producer, marking it as comparatively more tolerant. Near the MIC range (2–1.14 µg/mL), *S. pyogenes* showed the most consistent inhibition, whereas *A. baumannii*, *E. coli*, *S. aureus*, and *P. aeruginosa* shifted toward low-to-moderate biofilm production. At sub-MIC concentrations, the effect became species-dependent: *S. pyogenes*, *S. aureus*, and *E. coli* remained weak-to-moderate, while *A. baumannii* and *P. aeruginosa* reverted to strong biofilm near untreated phenotypes. These findings are consistent with a broad body of literature showing that AgNPs and other metal-based NPs reduce biofilm formation and sometimes disrupt established biofilms [26,30,34,35]. Electrochemically synthesized AgNPs reduced *P. aeruginosa* and *S. aureus* biofilm viability by up to >99.9% at multiples of the MIC and were shown by electron microscopy to deconstruct the extracellular matrix and damage bacterial membranes [26]. AgNP-loaded fungal textiles similarly inhibited *P. aeruginosa* biofilm formation *in vitro* and were non-toxic to *G. mellonella* larvae *in vivo*, supporting the translational potential of AgNP-based anti-biofilm strategies [36]. For phytochemical-capped or “green” AgNPs specifically, the antibiofilm mechanism is likely multifactorial and may differ from that of chemically or electrochemically synthesized counterparts due to the presence of the bioactive organic corona. In addition to the well-established AgNP-mediated pathways of membrane disruption, Ag^+^ release, and intracellular ROS generation [27,28,33], several lines of evidence suggest that the phytochemical capping layer contributes additional anti-biofilm functionality. First, phenolic compounds such as gallic acid, catechins, and flavonoid glycosides, all identified in the kombucha matrix used here, have been independently shown to interfere with bacterial quorum-sensing signaling, a central regulatory pathway for biofilm initiation and maturation [37]. Green-synthesized AgNPs capped with similar polyphenolic constituents have been demonstrated to suppress QS-regulated virulence factors and reduce biofilm development in *P. aeruginosa* at sub-MIC concentrations without affecting planktonic viability, indicating an anti-virulence rather than purely bactericidal mode of action [37]. Second, the polyphenol-rich corona may reduce bacterial adhesion to surfaces by modifying the physicochemical characteristics of both the nanoparticle and the substratum interface, thereby impeding the initial attachment step that precedes biofilm architecture formation [38]. Third, biogenic AgNPs have been reported to inhibit the production of extracellular polymeric substances (EPS), the structural scaffold of the biofilm matrix, through interference with the biosynthetic machinery downstream of quorum sensing [39]. The concentration-dependent anti-biofilm gradient observed in our data, with complete suppression at 40 µg/mL, sustained inhibition near the MIC, and species-dependent return toward untreated phenotypes at sub-MIC levels, is consistent with a threshold-driven mechanism in which the combined effects of Ag^+^ release and phytochemical QS interference must exceed a critical density to prevent biofilm establishment. The present work focused on biofilm prevention rather than the eradication of mature biofilms, which are typically more refractory to treatment [26,35]. Nevertheless, the observation that biofilm suppression can be maintained at near-MIC concentrations for several species and does not exceed untreated biofilm classifications at sub-MIC concentrations remains relevant, given that sub-inhibitory levels of conventional antibiotics often enhance biofilm production, for example, in *S. aureus* and *E. faecalis* [40,41].

The LC–MS/MS profiling of the rooibos kombucha fermentation matrix has direct implications for interpreting the functional properties of AgNPs-K. The T2 kombucha was enriched in gallic acid, protocatechuic acid, caffeic acid, chlorogenic acid, catechin/epicatechin, and EGCG, all of which are multi-hydroxylated phenolics with established redox activity and documented independent antimicrobial properties. Gallic acid and ferulic acid, both present in appreciable concentrations in the T2 kombucha, have been shown to disrupt bacterial membrane integrity by altering membrane permeability and surface hydrophobicity, causing local pore formation and leakage of intracellular constituents in *E. coli*, *P. aeruginosa*, *S. aureus*, and *L. monocytogenes* [42]. Similarly, EGCG, the major catechin detected in the kombucha matrix, has been demonstrated to inhibit the Tet(K) efflux pump in staphylococci at sub-MIC concentrations, increasing intracellular tetracycline accumulation and reversing resistance [43]. When these compounds form the capping corona of the AgNPs-K, they may act synergistically with the released Ag^+^ ions, providing a multi-target antimicrobial effect that could partly explain the very low MIC values observed (1.14–1.33 µg/mL). Similarly, the flavonoid glycosides identified in the matrix (isoquercitrin, rutin, vitexin, hyperoside) have documented antioxidant and anti-inflammatory properties that could contribute to the favorable *in vivo* safety profile observed in *G. mellonella*, potentially modulating the host response at the injection site and reducing nanoparticle-associated oxidative stress. With respect to antibiofilm activity, the phenolic acids and flavonoids in the corona may contribute to quorum-sensing interference and reduction of bacterial surface adhesion, as discussed above, providing mechanistic continuity between the phytochemical composition of the matrix and the biological activity of the final nanomaterial. While the present study did not formally isolate the contribution of individual phytochemicals from that of the silver core, the convergence between the identified corona composition and the functional profile of AgNPs-K supports the concept that the fermented kombucha matrix contributes not only to nanoparticle synthesis and stabilization, but also to the biological activity of the final product.

The toxicity studies in *G. mellonella* indicate a favorable *in vivo* safety profile for AgNPs-K. Across a wide concentration range, including undiluted AgNPs-K (400 µg/mL), larval injection did not increase mortality relative to Dulbecco’s phosphate-buffered saline (DPBS) over seven days, indicating good acute tolerability. This pattern aligns with prior reports that metal-based nanomaterials are well tolerated at biologically active exposures [44], as electrochemically synthesized AgNPs were non-toxic at concentrations effective against clinically relevant pathogens [26], and biogenic AgNPs tested against MDR *P. aeruginosa* showed no detectable toxicity even at high dosing [21], reinforcing that properly capped AgNPs typically fall within an acceptable safety window in this model. A comparable safety–efficacy balance has also been described for non-silver systems, with rosehip-functionalized Mg(OH)_2_ nanoparticles showing no mortality at an *in vivo* dose of 225 mg/kg and markedly improved survival in *S. aureus* infection [45].

When larvae were infected with bacterial pathogens and treated with AgNPs-K at species-specific MICs, survival improved significantly in six of seven models, with the largest benefit seen for Gram-negative infections (*A. baumannii*, *E. coli*, *K. pneumoniae*, and *P. aeruginosa*) and more modest but still significant protection for *Enterococcus* and *S. pyogenes*. The lack of statistically significant improvement in the *S. aureus* model after multiple-testing correction suggests species-specific differences in *in vivo* susceptibility that were not fully predicted by planktonic MICs alone. Similar patterns have been reported for AgC, with MBC concentrations significantly enhancing *G. mellonella* survival following MDR-EAEC infection, with survival rates approaching those of meropenem-treated larvae [25], and rosehip Mg(OH)_2_ NPs markedly increasing survival in *S. aureus*–infected larvae relative to infection controls [45]. Overall, the survival curves observed here fall within the range reported for other metal nanoparticles in *G. mellonella*, supporting the translational relevance of AgNPs-K as a potential therapeutic candidate. The differential *in vivo* performance against Gram-negative versus Gram-positive infections may partly reflect envelope architecture, as noted by Campo-Beleno et al. [21]. Once the Gram-negative outer membrane is compromised, Ag^+^ and nanoparticles penetrate readily into the periplasm and cytoplasm, whereas the thick Gram-positive peptidoglycan can act as a barrier to Ag^+^ diffusion. Our data support this, with Gram-negative survival curves showing the most pronounced separation between treated and untreated groups despite similar or slightly higher MICs. Host factors may also contribute, since immune activation by nanoparticles can vary across pathogens [16]. How kombucha-derived corona components interact with larval immunity remains to be dissected.

Methodologically, the present study follows core best-practice principles for using the *G. mellonella in vivo* infection model in metal nanoparticle research, including the use of last-instar larvae within a defined weight range, standardized injection sites and volumes, and replication across larval batches to reduce temporal and biological variability. These elements are widely emphasized as essential for improving reproducibility and cross-study comparability in nanoparticle toxicity and efficacy work using this model [16]. Such design choices strengthen confidence that the toxicity and therapeutic signals observed here reflect nanoparticle–host–pathogen interactions rather than batch-specific artifacts. At the same time, several limitations inherent to the *G. mellonella* system should be acknowledged. Although the larvae provide a robust innate immune platform with both cellular and humoral responses, they lack an adaptive immune system, and species-specific differences in pharmacokinetics, biodistribution, and long-term host responses may constrain direct extrapolation to vertebrate settings [46]. In addition, while survival remains a validated and widely used endpoint for first-line evaluation of antimicrobial nanoparticles in this model [47], it does not fully resolve whether improved outcomes arise from bacterial clearance, immune modulation, or broader physiological effects [48]. Recent synthesis of this literature highlights the added value of combining survival with bacterial burden, immune analysis, cytotoxicity indices, and histopathology to better map mechanism and safety boundaries [16]. Accordingly, future work should expand beyond survival to incorporate CFU enumeration from hemolymph or tissues, targeted immune profiling, oxidative stress markers, and histopathological assessment. This broader endpoint framework would help clarify how AgNPs-K mediate protection at the host–pathogen interface and would further strengthen the translational relevance of this promising kombucha-derived formulation.

## 4. Materials and Methods

### 4.1. Kombucha Tea Preparation

For the preparation of the fermented kombucha tea, the SCOBY (Symbiotic Culture of Bacteria and Yeast) starter was commercially acquired from a kombucha shop (Kombucha Now, LLC, Madison, WI, USA) [49]. Following, the fermented tea was brewed according to the following recipe: 1 L of filtered water was brought to a boil, and 20 g of organic rooibos (*Aspalathus linearis*; Demmers Teehaus, Cluj-Napoca, Romania) tea was added to the hot water and steeped for 5 min. The tea was then filtered to remove loose leaves, and 20 g of cane sugar was added and stirred until completely dissolved. The sweetened tea was then allowed to cool to 26 °C. Once the tea had cooled, the starter SCOBY and tea were added. The tea was then covered with a breathable cloth and secured with a rubber band. The tea was then placed in a warm, well-ventilated area out of direct sunlight, at 26 °C, and allowed to ferment for a minimum of 14 days before use in the biosynthesis of the NPs. Adequate maturity of kombucha tea was considered when the tea reached a pH of 2.5.

### 4.2. HPLC/LC–MS Identification and Quantification of Polyphenolic Compounds in the Kombucha Tea

The phytochemical profile of the kombucha tea was evaluated by LC–MS/MS using two previously validated analytical protocols. Analyses were performed on an Agilent 1100 HPLC Series system (Agilent Technologies, Santa Clara, CA, USA) coupled to an Agilent Ion Trap 1100 SL mass spectrometer (LC/MSD Ion Trap VL; Agilent Technologies, Santa Clara, CA, USA) [50,51,52].

The first LC–MS method targeted the identification and quantification of polyphenolic compounds. It employed a Zorbax SB-C18 column (100 mm × 3.0 mm, 3.5 μm; Agilent Technologies, Santa Clara, CA, USA) with a binary gradient composed of methanol and 0.1% acetic acid. Although the method was initially designed to analyze 28 polyphenolic standards, only a subset of these was detected and quantified in the analyzed extracts. Chromatographic conditions included a column temperature of 48 °C, a flow rate of 1 mL/min, and an injection volume of 5 μL. UV detection was carried out at 330 nm for phenolic acids and at 370 nm for flavonoids, while mass spectrometry operated in negative ESI mode [53].

A second LC–MS method was optimized for the detection of eight additional polyphenols: epicatechin, catechin, syringic acid, gallic acid, vanillic acid, protocatechuic acid, epigallocatechin, and epigallocatechin gallate. This method utilized the same chromatographic column and instrumentation, with a modified gradient elution scheme. Detection was conducted in MS mode under identical ESI conditions [53].

Compound identification was based on the comparison of MS spectra and chromatographic retention times with those of reference standards. Quantification was achieved via UV detection, using calibration curves constructed from analytical standards. Chromatographic data were processed using DataAnalysis (v5.3) and ChemStation (vB01.03) software (Agilent Technologies, Santa Clara, CA, USA), and results were expressed as micrograms of bioactive compound per milliliter of extract. Representative LC-DAD-MS chromatograms of the T0 and T2 samples are presented in Supplementary Figure S1.

### 4.3. Biosynthesis and Characterization of Silver Nanoparticles

The kombucha tea was used for the biosynthesis of AgNPs. A kombucha tea extract was first prepared by filtration using Whatman no.1 paper (Cytiva, Marlborough, MA, USA) until no visible biofilm strands were seen in the liquid. The tea was then filter-sterilized by successive filtration through a 0.45 µm filter followed by a 0.22 µm filter (Merck Millipore, Merck KGaA, Darmstadt, Germany). Lastly, the tea was diluted 1:10 with sterile distilled water and thus ready to use for NP biosynthesis. The silver ions from AgNO_3_ (Carl Roth GmbH + Co. KG, Karlsruhe, Germany) were reduced to their metallic form by adding 50 mL of kombucha tea extract dropwise into a 150 mL aqueous solution of 10^–3^ M of silver salt under vigorous stirring. The pH of the mixture was adjusted to 4.5 using NaOH solution (1 M; Carl Roth GmbH + Co. KG, Karlsruhe, Germany). The bio-reduction reaction started within a few minutes, as indicated by a significant color change from light yellow to deep red brown, confirming the formation of the AgNPs-K. After 3 h, the reaction was complete, and the obtained colloidal solution was further centrifuged at 6700× *g* for 10 min to separate the AgNPs-K pellet and washed with sterile distilled water. The washing process was repeated two times [54].

The AgNPs-K were characterized using various appropriate spectroscopic and microscopic techniques. UV-Vis spectroscopy was employed, using a Duetta™ Fluorescence and Absorbance Spectrometer (HORIBA Scientific, Kyoto, Japan) in the 300–800 nm wavelength range with a 2 nm resolution to confirm the presence of silver nanoparticles.

Nanoparticle tracking analysis (NTA) was performed with a ZetaView® instrument (S/N 17-328, Particle Metrix GmbH, Inning am Ammersee, Germany) and its proprietary ZetaView software (version 8.04.02), with a camera calibrated at 0.743 µm/pixel, for identification of nanoparticle Zeta potential and hydrodynamic size. The particle size distribution and morphology of the AgNPs-K were investigated by transmission electron microscopy using a H-7650 120 kV Automatic TEM Hitachi (Tokyo, Japan) on a carbon-coated copper grid.

Fourier transform infrared (FTIR) spectra of AgNPs-K and kombucha samples were recorded on a Perkin–Elmer Spectrum BX II spectrometer (Perkin–Elmer, Waltham, MA, USA) using the KBr pellet method. Solid samples (lyophilized AgNPs-K and kombucha solids) were finely ground and thoroughly mixed with dry spectroscopic-grade potassium bromide (Thermo Fisher Scientific, Waltham, MA, USA), then pressed into 7 mm pellets under 1.7 tons of pressure using a hydraulic press. Prior to each measurement, a background spectrum of a blank KBr pellet was collected under identical conditions and used as a reference. Spectra were acquired in absorbance mode over the 4000–400 cm^−1^ range, with a spectral resolution of 4 cm^−1^. A total of 32 scans were accumulated and averaged for both sample and background. The prepared KBr pellets were placed in the sample holder of the FTIR spectrometer, and the interferograms were collected and Fourier-transformed to obtain the final spectra. Spectral data were subsequently imported and processed in R version 4.5.1 (R Foundation for Statistical Computing, Vienna, Austria) for visualization and further analysis.

### 4.4. Antimicrobial Effects

#### 4.4.1. Determination of Minimum Inhibitory Concentration and Minimum Bactericidal Concentration

The antimicrobial properties of AgNPs-K were tested on *Pseudomonas aeruginosa* (ATCC BAA-47), *Klebsiella pneumoniae* (ATCC 700600), *Escherichia coli* (ATCC 25922), *Proteus mirabilis* (ATCC 7002), *Acinetobacter baumanii* (ATCC BAA-1605), *Staphylococcus aureus* (ATCC 25923), *Streptococcus pyogenes* (ATCC 19615), and *Enterococcus faecium* (ATCC 700221) by microdilution technique according to EUCAST methodology [55]. References strains were obtained from the American Type Culture Collection (ATCC, Manassas, VA, USA). An initial bacterial suspension was prepared by adding one/two 24 h bacterial colonies to 2 mL sterile saline, adjusting the turbidity to 0.4 McFarland. The microbial suspensions were further diluted in MHB (Mueller Hinton Broth; Oxoid Ltd., Basingstoke, UK) until a final inoculum of approximately 1 × 10^6^ CFU/mL was achieved. Following the preparation of the bacterial suspensions, 100 µL of the standardized bacterial inoculum (approximately 1 × 10^6^ CFU/mL) was added to the wells of a sterile 96-well microtiter plate (VWR International, Leuven, Belgium). For *Staphylococcus aureus*, cation-adjusted MHB broth (Mueller Hinton Broth; Oxoid Ltd., Basingstoke, UK) was used, while for *Streptococcus pyogenes*, MHB was supplemented with 5% defibrinated sheep’s blood (BioMaxima S.A., Lublin, Poland).

To test the effects of AgNPs-K and to verify that the antimicrobial activity was not attributable to residual kombucha components, serial dilutions of each substance were prepared in MHB. For each dilution, 100 µL of the diluted antimicrobial agent was added to the corresponding wells containing the 100 µL microbial suspensions, resulting in final concentrations of: 1/150, 1/200, 1/250, 1/300, 1/350, 1/400, 1/500, 1/750, and 1/1000 (equivalent to 2.66, 2, 1.6, 1.33, 1.14, 1, 0.8, 0.53, and 0.4 µg/mL). Positive control wells containing only microbial suspensions in MHB were included to ensure microbial viability, while negative control wells containing only MHB with AgNPs-K or kombucha filtrate were used to confirm the sterility and inherent turbidity of the antimicrobial agents. For each bacterial species, the assay was performed in four technical replicates and three biological replicates to ensure both statistical reliability and study replicability. The microtiter plates were covered and incubated at 37 °C for 24 h to allow for microbial growth and interaction with the test substances. After incubation, the plates were examined visually for turbidity and then further assessed by measuring the optical density at 620 nm (OD620) using a microplate reader (Multiskan MS microplate reader, Thermo Labsystems, Helsinki, Finland) to quantify microbial growth. The lowest concentration of AgNPs-K and kombucha tea that showed no visible growth, indicating an absence of turbidity, was recorded as the Minimal Inhibitory Concentration (MIC).

To verify the appropriate inoculum density and ensure accurate MIC determinations, a separate plating step was performed using a 1:20 dilution of the stock microbial suspensions. From this dilution, 100 µL was plated onto the appropriate agar plates. The plates were then incubated at 37 °C for 24 h, after which the colonies were counted. The aim was to achieve approximately 50 colonies per plate, which corresponds to an appropriate initial inoculum size for the microdilution assay. This step was crucial to confirm that the starting concentration of bacteria was consistent across all wells and within the expected range, providing confidence in the reliability of the assay results and the efficacy of the antimicrobial agents tested.

To determine the Minimal Bactericidal Concentration (MBC) of AgNPs-K, an MTT (3-(4,5-dimethylthiazol-2-yl)-2,5-diphenyltetrazolium bromide) assay was performed following the initial microdilution assay. MTT was acquired from Thermo Fisher Scientific (Waltham, MA, USA). After determining the MIC and identifying the wells with no visible growth, indicating potential MBC, 50 µL of an MTT solution (0.5 mg/mL) prepared in sterile phosphate-buffered saline (PBS; Fisher Scientific, Madrid, Spain) was added to each well of the microtiter plate. The plate was then covered and incubated at 37 °C for 2 h to allow viable cells to reduce the MTT to formazan, a purple-colored product. Following incubation, 150 µL of dimethyl sulfoxide (DMSO; Carl Roth GmbH + Co. KG, Karlsruhe, Germany) was added to each well to dissolve the formazan crystals. The plate was gently shaken for 10 min to ensure complete dissolution of the crystals. The absorbance of the resulting solution was then measured at 570 nm using a microplate reader. Wells showing no color change, indicating a lack of viable cells capable of reducing MTT, confirmed the MBC concentrations identified in the microdilution assay. For the MTT assay, all analyses were performed in quadruplicate to ensure statistical reliability. The absorbance readings were averaged, and the data were analyzed to determine the exact concentrations of AgNPs-K that resulted in no detectable microbial metabolic activity.

#### 4.4.2. Time-to-Kill Assay

Time–kill assays were performed for *Pseudomonas aeruginosa*, *Klebsiella pneumoniae*, *Escherichia coli*, *Proteus mirabilis*, *Acinetobacter baumannii*, *Staphylococcus aureus*, *Streptococcus pyogenes*, and *Enterococcus faecium* in a 96-well microtiter format matching the MIC setup. Each plate included a growth control (C+, bacteria + broth, no AgNPs-K) and a sterility control (C−, broth only). AgNP-K suspensions were combined with standardized inocula in organism-appropriate media to final concentrations of 1/150, 1/200, 1/250, 1/300, 1/350, 1/400, 1/500, 1/750, and 1/1000 (equivalent to 2.66, 2.00, 1.60, 1.33, 1.14, 1.00, 0.80, 0.53, and 0.40 µg/mL AgNPs-K). Cultures were incubated at 37 °C. At 0, 3, 5, 7, 9, 11, 15, 19, 21, and 24 h, 100 µL aliquots were removed, serially diluted, plated, and colonies were enumerated after 24 h at 37 °C. For each bacterial species, the assay was performed in four technical replicates and three biological replicates to ensure both statistical reliability and study replicability [56].

Raw bacterial colony counts were consolidated from time-stamped worksheets into a long/tidy dataset in R (v4.5.1) using readxl and tidyverse, and CFU/mL were computed as

$$CFU/mL=\frac{\mathrm{colonies}}{\mathrm{volume}\;\mathrm{plated}\;(\mathrm{mL})}\times \frac{1}{\mathrm{dilution}\;\mathrm{plated}},$$

with the experiment-specific dilution (10^−4^). For each time × concentration, we calculated the mean (and SD, *n*), and log_10_(CFU/mL). Control series (C+, C−) were summarized at the same timepoints.

Because the growth control (C+) reflects concurrent replication in the same medium, all effects were referenced to C+ at the same time point. We defined the instantaneous effect as

$$\Delta {log}_{10}(t)={log}_{10}[CFU/mL{]}_{C+}(t)-{log}_{10}[CFU/mL{]}_{\mathrm{dose}}(t).$$

Classification at the endpoint was

- Bactericidal: $\Delta {log}_{10}(24\;h)\geq 3.00$ (≥99.9% relative reduction vs. C+).
- Bacteriostatic: $0.00<\Delta {log}_{10}(t_{\mathrm{end}})<3.00$ (reduction vs. C+, but <3 log).
- No net inhibition/growth: $\Delta {log}_{10}(t_{\mathrm{end}})\leq 0.00$ (equal to or worse than C+).

For each concentration, we estimated the time to achieve Δlog_10_ = 0.301, 1.000, and 3.000 (T_50_, T_90_, T_99.9_) relative to C+. Two complementary R approaches were used:

A. Observed-curve interpolation (used for figures/markers and robustness): 

We constructed continuous functions of the observed mean log_10_(CFU/mL) for C+ and for each dose using stats::approxfun. We then computed $\Delta {log}_{10}(t)$ on a fine time grid and obtained T-metrics by linear interpolation at the target thresholds (0.301/1.000/3.000).

B. Model-based fits (used for summary tables when fits converged): 

Time–kill trajectories for C+ and for each dose were fit by a one-phase exponential with a Plateau using minpack.lm::nlsLM:

$$y(t)=\mathrm{Plateau}+(Y_{0}-\mathrm{Plateau})\;e^{-kt},$$

with $y(t)={log}_{10}(CFU/mL)$. Initial values were derived from the data (mean at *t* = 0 for $Y_{0}$, time-series median for *Plateau*, starting k≈0.2). T-metrics were then obtained by root-finding (uniroot) on the fitted $\Delta {log}_{10}(t)$ curves (C+ minus dose). If a model did not converge or the target was not reached within the observed window, T was reported as NA.

For each isolate and concentration, the net change at prespecified times was summarized as $\Delta {log}_{10}(t)$ relative to C+. Where dose–response modeling was of interest, a three-parameter Hill model with bottom fixed to 0 was optionally fit in R to $\Delta {log}_{10}(t)$ vs. concentration to obtain Emax, EC_50_, and EC_90_ (nonlinear least squares). If model assumptions were not satisfied, we reported non-parametric summaries (medians/IQR) of $\Delta {log}_{10}(t)$ across concentrations.

All data analyses and plots were performed in R version 4.5.1 using tidyverse, readxl, broom, minpack.lm, and ggplot2.

#### 4.4.3. Biofilm Quantification Assay

To quantify the inhibition of bacterial biofilm formation by biosynthesized nanoparticles, strong biofilm-producing strains of the following bacteria were used: *Pseudomonas aeruginosa* (ATCC BAA-47), *Klebsiella pneumoniae* (ATCC 700600), *Escherichia coli* (ATCC 25922), *Acinetobacter baumannii* (ATCC BAA-1605), *Staphylococcus aureus* (ATCC 25923), and *Streptococcus pyogenes* (ATCC 19615). To assess the potential inhibitory effect of biosynthesized silver nanoparticles, a biofilm quantification assay was performed using crystal violet staining in a 96-well microtiter plate format. The protocol was adapted from Stepanović et al. [57]. Overnight cultures of Gram-negative bacteria were prepared in Mueller–Hinton broth supplemented with 1% glucose, while Gram-positive bacteria were cultured in brain heart infusion broth (BHI; Oxoid Ltd., Basingstoke, UK) with 1% glucose to enhance biofilm production. All bacterial suspensions were adjusted to an inoculum of approximately 1 × 10^8^ CFU/mL.

To conduct the biofilm assay, 100 µL of each microbial suspension was added to the wells of a sterile 96-well microtiter plate. For each bacterial species, the assay was performed in eight technical replicates and three biological replicates to ensure both statistical reliability and study replicability. To assess the effects of AgNPs-K, 100 µL of each test substance at predetermined concentrations 1/10, 1/100, 1/200, 1/250, 1/350, 1/500, 1/750, 1/1000, and 1/1250 (equivalent to 40, 4, 2, 1.14, 0.8, 0.53, 0.4, and 0.32 µg/mL) was added to the corresponding wells containing microbial cultures. Wells containing only microbial suspensions without any test substance served as positive controls, while wells with only the test substances and sterile broth served as negative controls. The plates were incubated at 37 °C for 24 h, maintaining the humidity saturation conditions to allow for biofilm formation. Following the incubation, the wells were gently washed three times with PBS to remove non-adherent planktonic cells, leaving only the biofilm attached to the well surfaces. The plates were then air-dried at room temperature for 24 h to ensure complete dehydration of the biofilms. After air drying, the biofilms were fixed by adding 200 µL of methanol (Carl Roth GmbH + Co. KG, Karlsruhe, Germany) to each well and allowing it to sit for 20 min. The methanol was then carefully removed, and the plates were left to air dry again for 24 h to ensure proper fixation of the biofilm matrix.

To quantify the biofilm biomass, 200 µL of 0.1% crystal violet solution (Carl Roth GmbH + Co. KG, Karlsruhe, Germany) was added to each well, and the plate was incubated for 30 min at room temperature to stain the biofilm. Excess stain was removed by washing the wells three times with PBS. The bound crystal violet was eluted by adding 200 µL of 0.2 M hydrochloric acid (HCl) to each well, followed by a 30-min incubation at room temperature to ensure complete elution of the stain. The absorbance of the eluted crystal violet was measured at 570 nm using a microplate reader to quantify the biofilm biomass in each well. The absorbance values were directly proportional to the amount of biofilm present. A reduction in absorbance in wells treated with AgNPs-K, compared to the positive control wells, indicated inhibition of biofilm formation. Data from quadruplicate wells were averaged, and statistical analyses were performed to determine the significance of the inhibitory effects of AgNPs-K on biofilm formation. Considering a low cut-off (ODc) represented by 3 × SD above the mean OD of negative control wells, strains were classified as no biofilm producer (OD ≤ ODc), weak biofilm producer (ODc < OD ≤ 2 × ODc), moderate biofilm producer (2 × ODc < OD ≤ 4 × ODc), or strong biofilm producer (4 × ODc < OD) [57].

### 4.5. In Vivo Experiments Using Galleria mellonella Larvae

*Galleria mellonella* was selected as the in vivo model because it combines a well-characterized innate immune system, with both cellular and humoral responses functionally analogous to vertebrate innate immunity, with the ability to be maintained at 37 °C, the physiologically relevant temperature for human pathogens. The model also permits standardized hemocoel injection of both pathogen and treatment, allowing precise dose control [14,15]. As an invertebrate not covered by EU Directive 2010/63/EU, *G. mellonella* requires no ethical approval and provides an ethically favorable intermediate step between *in vitro* assays and vertebrate studies. Its suitability for evaluating metal nanoparticles has been specifically endorsed by recent systematic reviews and best-practice guidelines for this research area [16].

#### 4.5.1. Procurement and Selection of *Galleria mellonella* Larvae

**Figure 14:**
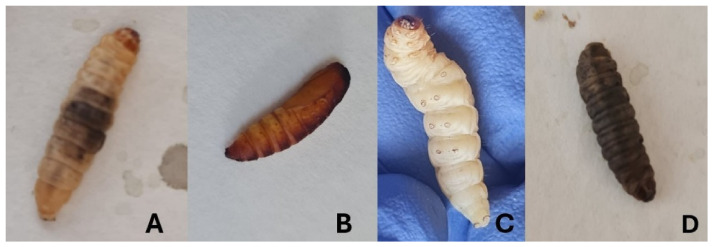
Examples of larvae excluded from the study. (**A**) Melanization on *Galleria mellonella* larvae. (**B**) Cocooned larvae. (**C**) Larvae with abnormality (extra proleg). (**D**) Dead larvae.

Larvae were purchased from a Romanian vendor from Cluj-Napoca, Cluj County [58] and transported to the Microbiology Discipline of “Iuliu Hatieganu” University of Medicine and Pharmacy and used for *in vivo* experiments the same week. Last instar larvae were selected, with a weight between 300 and 400 mg. Larvae that presented signs of disease or melanization were not included in the study (Figure 14). Larvae were housed in sterile plastic Petri dishes, with wood shavings as bedding, at room temperature (24 °C), in the dark.

#### 4.5.2. Acute Nanoparticle Toxicity in *G. mellonella* Larvae

The external surface of the larvae was sterilized with 70% ethanol, followed by injection using a Hamilton Gastight 1705 RNR syringe (Hamilton, Reno, NV, USA) through the last left proleg. A 10 μL inoculum containing AgNPs-K diluted in PBS at the indicated concentrations (undiluted stock, 1/10, 1/100, and 1/1000, equivalent to 400, 40, 4, and 0.4 µg/mL) was administered. PBS was selected as the AgNPs-K diluent rather than DPBS because the divalent cations present in DPBS compress the electrical double layer of biogenic AgNPs and promote aggregation, compromising nanoparticle stability prior to injection [59]. Control larvae received 10 μL DPBS (Fisher Scientific, Madrid, Spain), the standard vehicle control for *G. mellonella* injection studies. After injection, larvae were incubated at 37 °C for 7 days, with mortality assessed every 24 h post-injection. Mortality was defined as the absence of movement upon tactile stimulation and the presence of dark pigmentation on the cuticle. Survival curves were generated using Kaplan–Meier analysis, and statistical significance was evaluated using the log-rank test (Prism 11, GraphPad Software, San Diego, CA, USA). Each group consisted of 10 larvae, and the experiment was conducted in triplicate (n = 30). Larvae were randomly assigned to groups using a randomization software [60]. The experiment was repeated two months later to account for potential phenotypic variation between larval batches and to minimize seasonal effects on larval immune responses.

#### 4.5.3. *G. mellonella* Bacterial Infection Model and Treatment with AgNPs-K

The external surface of larvae was disinfected with 70% ethanol. For infection, a 0.4 McFarland bacterial suspension (10 μL) was injected intra-haemocoelically into the last left proleg using a Hamilton Gastight 1705 RNR syringe. A negative control group received 10 μL DPBS, and a nanoparticle-only control group received AgNPs-K at the species-specific MIC value (1.14 µg/mL for Gram-positive species and 1.33 µg/mL for Gram-negative species). Each group contained 10 larvae, and allocation was randomized using a computer-generated sequence [31]. The experiment was repeated two months after the initial run to minimize phenotypic differences between larval batches and potential seasonal variation in larval immunity (total n = 30).

**Figure 15:**
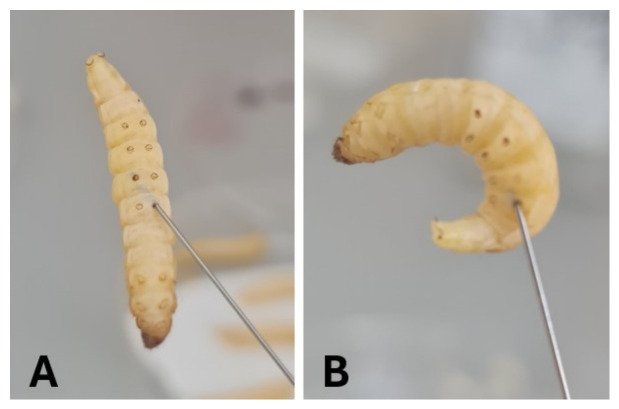
Image depicting injection sites of *Galleria mellonella* last instar larvae. (**A**) Treatment injection site. (**B**) Bacterial inoculum injection site.

Treatment groups received AgNPs-K at the species-specific MIC (1.14 µg/mL for Gram-positive species and 1.33 µg/mL for Gram-negative species), administered as a 10 µL intra-haemocoelic injection through the last right proleg, 30 min after bacterial inoculation through the last left proleg (Figure 15). This post-infection treatment design was chosen to evaluate the therapeutic rather than prophylactic activity of AgNPs-K, with the 30-min interval allowing initial bacterial dissemination within the hemocoel before treatment delivery. After injection, the larvae were placed in Petri dishes and incubated at 37 °C. The larvae were monitored daily for seven consecutive days. Mortality was determined by the absence of movement in response to touch and the presence of dark pigmentation on the cuticle. For survival analysis, daily counts of larvae alive were converted to individual time-to-event records (deaths recorded on the first day observed; larvae alive on day 7 were right-censored). Kaplan–Meier survival curves were generated, and group differences were tested using the log-rank (Mantel–Cox) test. In addition to the overall log-rank across groups, pairwise log-rank comparisons were performed with Holm-adjusted for multiple testing. Analyses and graphics were produced in R version 4.5.1 using the survival and survminer packages. Where shown, significance on plots is annotated as *p* < 0.05 (*), *p* < 0.01 (**), and *p* < 0.001 (***), based on Holm-adjusted pairwise log-rank *p*-values.

## 5. Conclusions

Taken together, our findings support several broader implications. First, they reinforce the concept that the complex phytochemical matrix of fermented rooibos kombucha can be harnessed not only to generate stable silver nanoparticles but also to endow them with potent and broad-spectrum antimicrobial properties at very low concentrations. Second, they show that such nanoparticles can strongly inhibit biofilm formation, a critical feature given the central role of biofilms in chronic, device-associated, and treatment-refractory infections. Third, they demonstrate that AgNPs-K are non-toxic and efficacious in an invertebrate infection model, supporting the value of *Galleria mellonella* as a practical intermediate step between *in vitro* screening and vertebrate studies. In light of the escalating burden of antimicrobial resistance, kombucha-derived silver nanoparticles warrant further exploration in mammalian systems, including detailed toxicological profiling, pharmacodynamic evaluation, and testing in clinically relevant planktonic and biofilm-driven infection models.

## Figures and Tables

**Table 1:** HPLC analysis of the kombucha fermented tea.

Compound	Concentration (µg/mL)	
	T0	T2
Gentisic acid	<LOQ	<LOQ
Caffeic acid	<LOQ	1.046
Chlorogenic acid	<LOQ	1.383
4-O caffeoylquinic acid	1.32	2.162
p-coumaric acid	1.223	1.103
Ferulic acid	7.939	4.602
Vitexin	6.383	5.84
Hyperoside	5.587	5.69
Vitexin 2-O-Rhamnoside	ND	ND
Isoquercitrin	14.991	14.374
Rutoside (rutin)	5.162	6.201
Quercitrin	<LOQ	<LOQ
Quercetol	2.156	1.55
Luteolin	1.435	0.951
Epicatechin	1.57	1.514
Catechin	0.25	0.314
Syringic acid	0.645	0.557
Gallic acid	2.287	7.039
Protocatechuic acid	12.1	12.774
Vanillic acid	3.156	2.558
Epigallocatechin (EGC)	ND	ND
Epigallocatechin gallate (EGCG)	7.047	3.863

T0 = rooibos tea with added SCOBY prior to fermentation. T2 = rooibos tea with SCOBY after two weeks of fermentation. <LOQ = value below quantification; ND = not determined.

**Table 2:** Effects of different concentrations of AgNP-K on bacterial biofilm production.

	Bacterial Biofilm Production Under Treatment with Different AgNP-K Concentrations (µg/mL)									Positive Control
Bacterial strain	40	4	2	1.6	1.14	0.8	0.53	0.4	0.32	
*Streptococcus* *pyogenes*	None	None	None	None	None	Weak	Moderate	Moderate	Moderate	Moderate
*Staphylococcus* *aureus*	None	None	Weak	Weak	Moderate	Moderate	Moderate	Moderate	Moderate	Moderate
*Acinetobacter* *baumannii*	None	None	None	Weak	Weak	Weak	Moderate	Moderate	Strong	Strong
*Escherichia* *coli*	None	None	None	Weak	Weak	Weak	Weak	Moderate	Moderate	Moderate
*Klebsiella* *pneumoniae*	None	Weak	Moderate	Moderate	Moderate	Moderate	Moderate	Moderate	Moderate	Moderate
*Pseudomonas* *aeruginosa*	None	None	Weak	Weak	Moderate	Moderate	Strong	Strong	Strong	Strong

## Data Availability

The original contributions presented in this study are included in the article/Supplementary Materials. Further inquiries can be directed to the corresponding author.

## Supplementary Materials

The following supporting information can be downloaded at https://www.mdpi.com/article/10.3390/ijms27125274/s1.

## Abbreviations

The following abbreviations are used in this manuscript:

AgNPs	silver nanoparticles
AgNPs-K	kombucha-synthesized silver nanoparticles
AgNO_3_	silver nitrate
ATCC	American Type Culture Collection
BHI	brain heart infusion
CFU	colony-forming units
CV	crystal violet
DPBS	Dulbecco’s phosphate-buffered saline
DMSO	dimethyl sulfoxide
EGCG	epigallocatechin gallate
EUCAST	European Committee on Antimicrobial Susceptibility Testing
FTIR	Fourier-transform infrared spectroscopy
HPLC	high-performance liquid chromatography
LC–MS/MS	liquid chromatography–tandem mass spectrometry
MBC	minimum bactericidal concentration
MHB	Mueller–Hinton broth
MIC	minimum inhibitory concentration
MTT	(3-(4,5-dimethylthiazol-2-yl)-2,5-diphenyltetrazolium bromide) assay
NTA	nanoparticle tracking analysis
OD	optical density
ODc	optical density cut-off
PBS	phosphate-buffered saline
ROS	reactive oxygen species
SCOBY	symbiotic culture of bacteria and yeast
SPR	surface plasmon resonance
TEM	transmission electron microscopy
UV–Vis	ultraviolet–visible spectroscopy
