# Supplementary material for: Silver Nanoparticles Produced by Rooibos Kombucha Suppress Bacterial Biofilms and Improve Survival in Galleria mellonella Infection Model
Source: Int J Mol Sci. 2026 Jun 10;27(12):5274. doi: 10.3390/ijms27125274 (PMC13299197; doi:10.3390/ijms27125274)
Supplement: Supplementary file 1 [file ijms-27-05274-s001.zip › ijms-4326673-supplementary.pdf]

Kombucha - T0

UV chromatogram (black) and MS total ion chromatogram (blue)

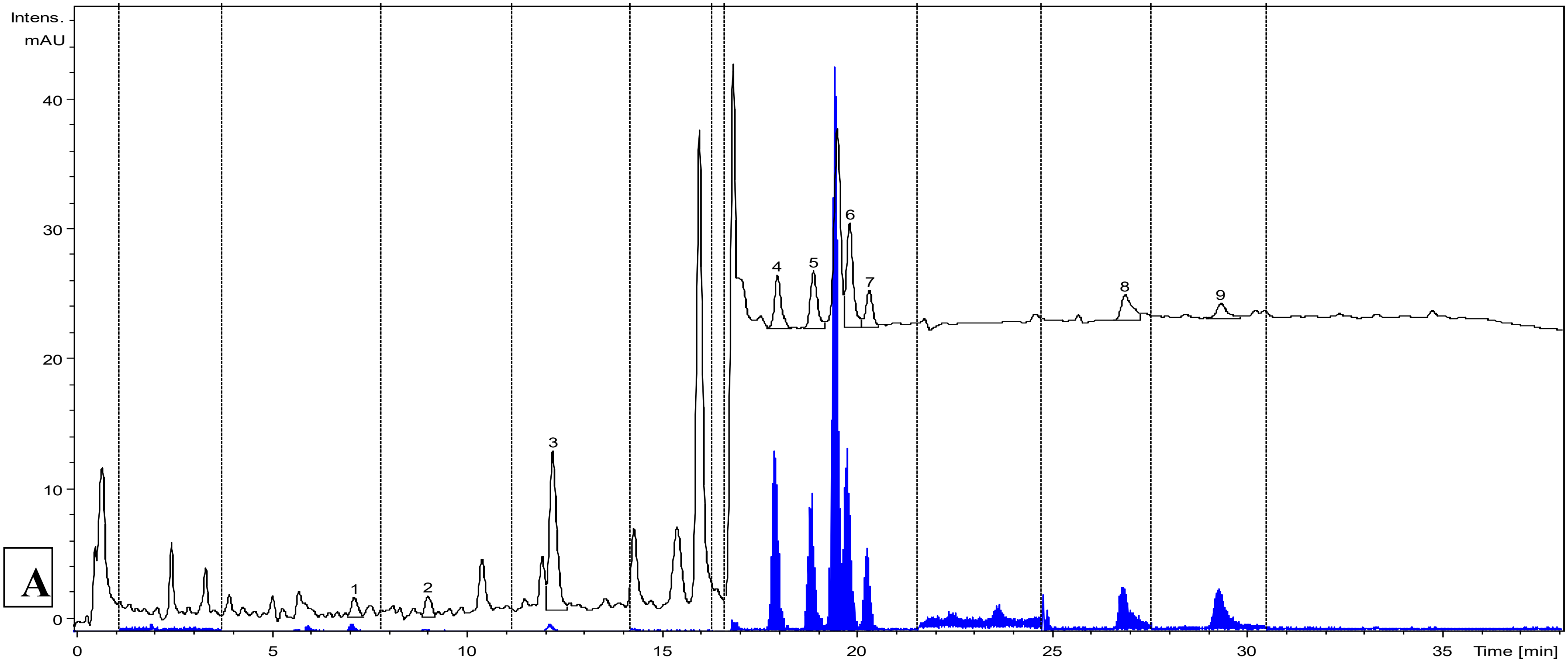

Kombucha – T2

UV chromatogram (black) and MS total ion chromatogram (blue)

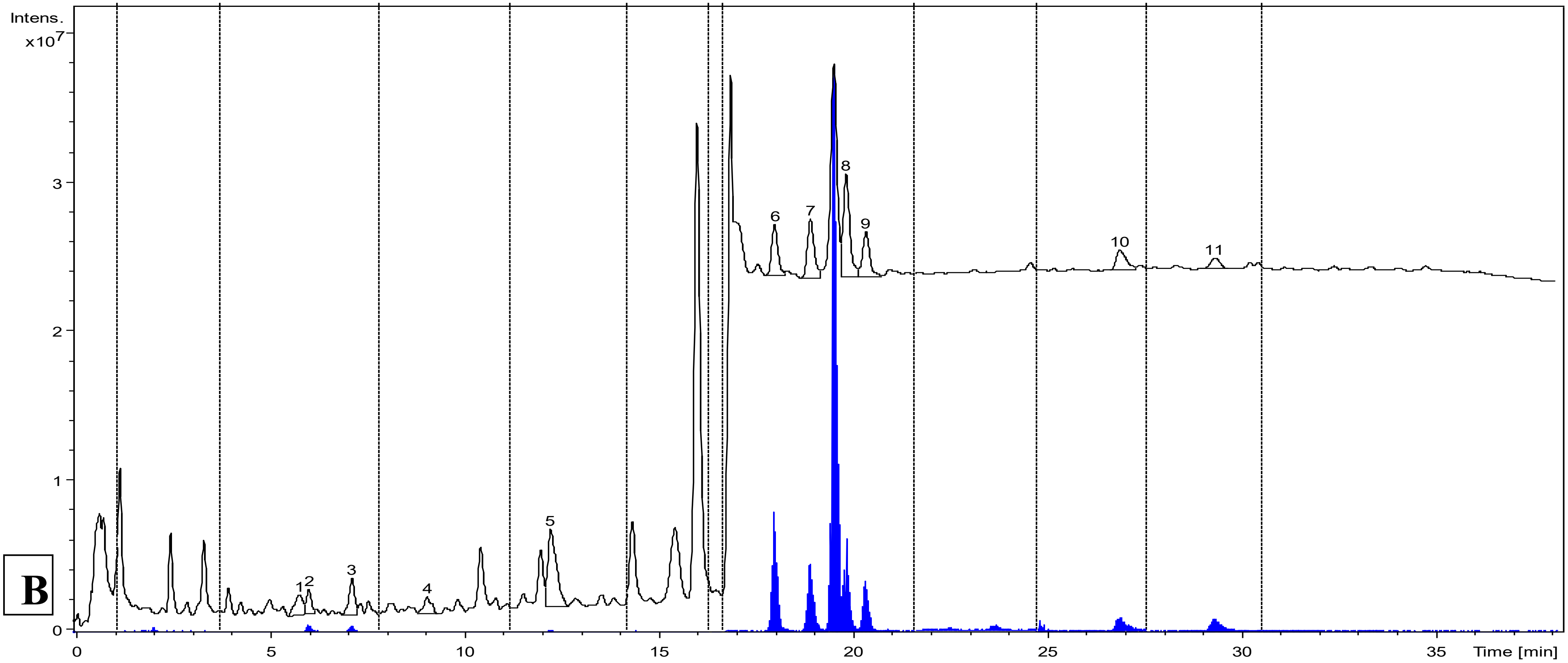

Figure S1. LC-DAD-MS chromatograms of rooibos kombucha before fermentation (T0) and at the endpoint of the 14-day fermentation (T2). **(A)** T0 sample. **(B)** T2 sample. In both panels, the black trace is the UV-DAD chromatogram and the blue trace is the total ion chromatogram acquired in negative electrospray ionization mode. Numbered peaks indicate the integration regions used for the major phytochemical constituents identified by tandem mass spectrometry. Peak numbering is independent between the two panels and reflects the chronological order of elution within each chromatogram. The chromatograms illustrate the qualitative redistribution of phenolic compounds during fermentation, including the appearance of two additional integrated regions in the T2 sample (peaks 10 and 11) and altered relative intensities of the polyphenolic constituents eluting between approximately 17 and 21 minutes.
